# Comparison of the effect of three autogenous bone harvesting methods on cell viability in rabbits

**DOI:** 10.15171/joddd.2017.014

**Published:** 2017-06-21

**Authors:** Janet Moradi Haghgoo, Seyed Reza Arabi, Seyyed Mohammad Hosseinipanah, Ghasem Solgi, Neda Rastegarfard, Maryam Farhadian

**Affiliations:** ^1^Department of Periodontics, Faculty of Dentistry, Hamadan University of Medical Sciences, Hamadan, Iran; ^2^Department of Anatomical Science, Faculty of Medicine, Hamadan University of Medical Sciences, Hamadan, Iran; ^3^Department of Immunology, Faculty of Medicine, Hamadan University of Medical Sciences, Hamadan, Iran; ^4^Modeling of Noncommunicable Diseases Research Center, Department of Biostatistics, Faculty of Public Health, Hamadan University of Medical Sciences, Hamadan, Iran

**Keywords:** Autograft, autologous transplants, dental Instruments, piezosurgery, tissue harvesting

## Abstract

***Background.*** This study was designed to compare the viability of autogenous bone grafts, harvested using different methods, in order to determine the best harvesting technique with respect to more viable cells.

***Methods.*** In this animal experimental study, three harvesting methods, including manual instrument (chisel), rotary device and piezosurgery, were used for harvesting bone grafts from the lateral body of the mandible on the left and right sides of 10 rabbits. In each group, 20 bone samples were collected and their viability was assessed using MTS kit. Statistical analyses, including ANOVA and post hoc Tukey tests, were used for evaluating significant differences between the groups.

***Results.*** One-way ANOVA showed significant differences between all the groups (P=0.000). Data analysis using post hoc Tukey tests indicated that manual instrument and piezosurgery had no significant differences with regard to cell viability (P=0.749) and the cell viability in both groups was higher than that with the use of a rotary instrument (P=0.000).

***Conclusion.*** Autogenous bone grafts harvested with a manual instrument and piezosurgery had more viable cells in comparison to the bone chips harvested with a rotary device.

## Introduction

One of the most common problems in the treatment of periodontal diseases is bony defect that compromises the prognosis of the treatment.^1-3^ In these situations the most favorable result is achieved by using reconstructive treatment.^3,4^ Hegedus in 1923 was the first to use autogenous bone grafts for reconstruction of bony defects.^5^ Currently the gold standard graft for reconstructing bone defects is still autogenous bone despite the introduction of different groups of bone substitutes.^6-8^ The best advantage of an autograft is maintaining the viability of the transplanted cells.^9-12^ However, various factors can affect the viability, including the method used for bone harvesting.^12^ Different techniques and tools are available for bone harvesting. Use of manual instruments such as chisel or rongeur is the conventional method that cause minimal thermal and mechanical trauma to the bone. However, usually the harvesting procedure takes a long time. The most common method for bone cutting is to use rotary devices^10-13^ that produce macrovibration, leading to more damage to bone structures and cells.^14-16^ Recently, with respect to the general trend toward less invasive and more accurate and safer surgical techniques, piezosurgery was introduced by Vercellotti.^14,17^ In this technique osteotomy is performed by ultrasonic vibration. The characteristic features of bone cutting are minimal surgical trauma, a desirable control during surgery and a rapid healing response.^14,16,17^

Selecting a precise and safe technique for bone cutting is probably the most important factor in maintaining the viability of osteoblast cells.^16^ In spite of the widespread use of piezosurgery in osteotomy, the outcomes of studies have not shown further definitive advantages of this method.^15^ The results of some studies support the superiority of piezosurgery in bone harvesting,^12,18^ while there are studies that have shown no further advantages for bone harvesting by piezosurgery.^10,19,20^ Furthermore, the available studies are mostly based on the histological evaluation of the grafts and the responses of the graft cells after being cultured.^11,18,21^ In addition, a small number of studies have compared the viability of graft cells by assessing the metabolically active live cells.^10,11^ Therefore the aim of the present study was to compare the effect of three harvesting methods, including piezosurgery, rotary devices and hand instruments, on cell viability of bone grafts.

## Methods

Ten white male New Zealand rabbits, 1–2 years of age and weighing between 1.5 and 2.5 kg, were included in the present study. Ethical approval for the experiment was obtained from the Ethics Committee in Animal Research of Hamadan University of Medical Sciences under the code IR.UMSHA.REC.1393.10.214. The animals were kept under standard conditions of animal laboratory for at least two weeks prior to the start of the experiment. Twenty bone graft samples were obtained for each study group. Bone samples were collected from the lateral body of the mandible on the left and right sides and in each side 3 different bone samples were harvested. As the quality of bone could affect the amount of viable cells of the graft, the sites of harvesting bone with each three different techniques were respectively changed in each side of the mandibular body.

**Figure 1:**
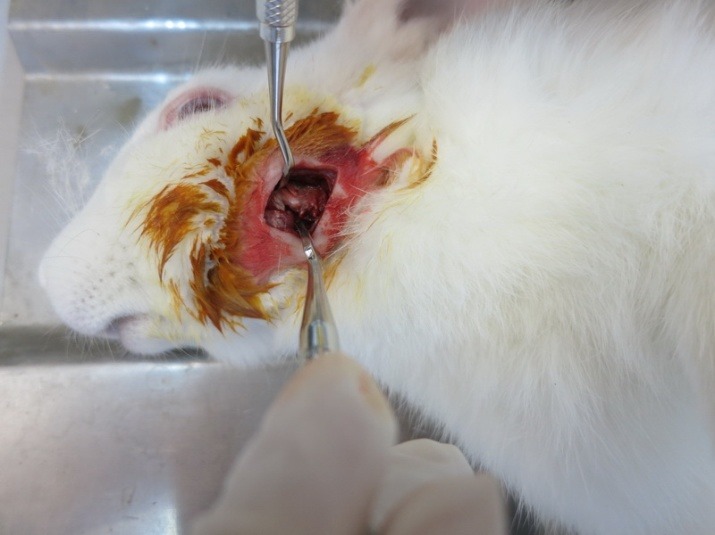
Exposure of the lateral part of mandibular body by a submandibular incision.

**Figure 2:**
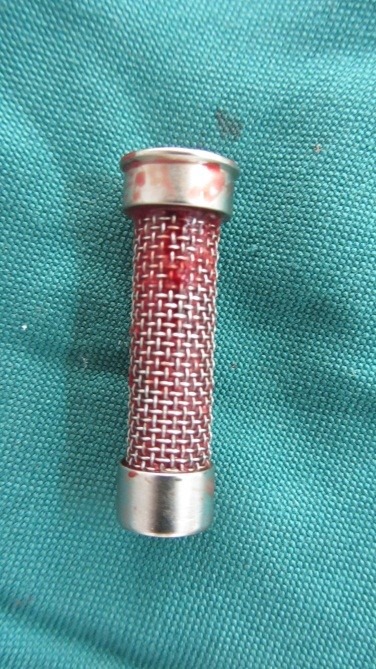
Filter of KOHLER suction tube for collecting harvested bone grafts

The surgical procedures of bone harvesting in all the animals were performed by a postgraduate student of periodontology. The animals were anesthetized using intramuscular injection of 2% xylazine (5 mg/kg) and 10% ketamine (40 mg/kg). The lateral part of the mandibular body was exposed with a submandibular incision (Figure 1). Three bone samples were harvested, measuring 3×3 mm in dimension and 4 mm in depth with a distance of 5 mm from the adjacent harvested area. The methods of bone harvesting included the followings:

1. Using a manual instrument, JOVANOVIC mini periodontal chisel, 2-mm (Hu-Friedy, Chicago, IL, USA) bone grafts were harvested.
2. Using a low-speed handpiece at a speed of 600 rpm (NSK, Shinagawa, TKY, Japan) by a round bur with a 2.2-mm diameter and with continuous irrigation and intermittent pressure during cutting, bone grafts were harvested and collected with a bone trap filter inserted at the suction tip (Figure 2).
3. WUsing piezosurgery (piezoelectric device, NSK, Shinagawa, TKY, Japan) particulated bone grafts were harvested and collected with a bone trap filter. The OP1 tip was used with relatively light strokes and a low pressure was applied to the handpiece. The surgery was performed under intermittent pressure during cutting, along with water spray for reducing thermal injuries.

The collected bone grafts were kept in a conditioned media and transferred to the laboratory for evaluating cell viability. Finally the incision was sutured and the animals were treated with antibiotics for 5‒7 days, including subcutaneous enrofloxacine (0.7 mL/day) and flurbiprofen (0.3 mL/day).^22^

In order to assess cell viability, the MTS kit (3,4,5-dimethylthiazol 2,5-diphenyltetrazolium bromidese) was used. Enzyme-based methods using MTS are easy-to-use and safe, with a highly reproducible, quantitative and reliable assay for determining the number of viable cells in a given culture. MTS is a sensitive method for evaluating osteoblast viability by oxidation of MTS with mitochondrial dehydrogenase. As a result of the reduction of yellow tetrazolium salt of MTS with mitochondrial dehydrogenase, purple crystals of formazan are formed. The amount of the purple product is proportional to the number of living cells in the sample and is determined by measuring the photometric absorption of the colorful solution. The laboratory steps of the test included the following: 100 mg of the fresh samples of the harvested bone grafts were washed with PBS to remove any unattached cells; then the samples were incubated with MTS (Promega, Madison, WI, USA) that was buffered with 400 µL of phosphate solution for 3 h at 37˚C in 5% CO_2_ incubator. Cell viability was evaluated with ELISA reader (Sunrise^TM^ Microplate Reader, Switzerland) at 490 nm.

## Result

Descriptive statistics, including means and standard deviations for viability of osteoblasts cells evaluated with ELISA Reader at 490 nm, were calculated in all the study groups. It was 48.80±4.05% for manual instrument group, 39.64±5.40% for rotary device group and 49.92±5.09% for piezosurgery group (Table 1). Then, the results were analyzed with one-way ANOVA. Statistical significance was set at P<0.05. One-way ANOVA showed significant differences between all the groups (P=0.000). The results of two-by-two comparisons of the study groups with post hoc Tukey tests revealed significant differences between the manual instrument and the rotary device (P=0.000) and piezosurgery and the rotary device (P=0.000); however, the manual instrument and piezosurgery had no significant differences (P=0.749) (Table 2).

**Table 1:** The mean cell viability in study groups (%)

**Bone harvesting method**	** N**	**Mean ± SD (%)**
**Manual instrument**	20	48.80±4.05
**Rotary device**	20	39.64±5.40
**Piezosurgery**	20	49.92±.5.09

**Table 2:** The results of two-by-two comparisons of study groups

**Bone harvesting method**	**Manual instrument**	**Rotary device**	**Piezosurgery**
**Manual instrument**	-	9.165^*^	-
**Rotary device**	-	-	
**Piezosurgery**	1.120	10.285^*^	-

* Statistically significant (P <0.05)

## Discussion

Autogenous bone grafts are the ideal choice for bone grafting, mainly because of osteoinductivity, osteogenicity and osteoconductivity properties and the possibility of having living bone cells.^1-3^ However, the viability of the transplanted cells of bone graft can be affected by several factors such as the method used for harvesting the graft, which is in concordance with the damage induced to the bone structure and cells during the harvesting procedure.^12^ Therefore, application of a method that minimizes the trauma to the bone would be effective in maintaining the cell viability.

In this study, analysis of cell viability in manual instrument, rotary device and piezosurgery groups using MTS kit showed that in all the groups, the cell viability was preserved. Gruber et al,^9^ Chiriac et al,^20^ Miron et al^10^ and Kuttenberger et al^23^ also showed that cell viability of the harvested autogenous bone grafts could be preserved.^9,10,20,23^ The highest cell viability was seen in piezoelectric and manual instrument groups. Consistent with the results of the present study, Berengo et al,^21^ Von see et al^12^ and Pekovits et al^18^ did not show a significant difference in cell viability between the groups of manual instrument and piezoelectric device.^12,18,21^ The lowest cell viability was observed in the rotary group, consistent with the results of Von See et al.^12^ However, in these studies cell viability was determined by culturing osteoblast cells and evaluating the proliferation and differentiation responses. Thus the primary cells of the harvested grafts were not compared.^12,18,21^ However, in the present study viability of the cells was evaluated by assessing the enzymes of active live cells with MTS kit, which suggested that it could better represent cell viability.^17^ The better results of manual method could be explained by the lower risk of thermal and mechanical trauma. In addition, because there is no need for washing the surgical site, the obtained bone grafts will be immersed in blood. This situation suggested that it could better preserve the cell viability.^12,18,20^ In piezosurgery the bone is cut with little pressure by micromechanical vibration that needs little energy; thus little heat is generated. In addition, it minimizes the trauma to the hard tissue by a micrometric bone cutting; therefore, it creates a precise bony preparation and better preserves the bone structures and cells. In addition, it has been shown that micromechanical cutting of bone leads to realization of bone morphogenic proteins that are favorable for the process of bone formation.^14,16,17^ As a result of these factors, the surgery can be carried out with minimal trauma; therefore, it is expected to improve cell viability. However, using rotary systems for bone cutting produces macrovibration that causes lamellar bone fracture and more thermal damage; therefore, lower viable cells would be maintained.^14-16^

In contrast to the results of the present study, Chiriac et al,^20^ Tete et al,^24^ Miron et al,^19^ Bacci et al^25^ and Miron et al^10^ showed the same osteogenic potential for piezoelectric and rotary methods and regarding the osteogenic potential, the manual instrument was preferred.^10,19,20,24,25^ In these studies, different methods were used for evaluating the osteogenic potential of autogenous bone grafts.^19,20,24,25^ Only Miron et al^10^ evaluated cell viability through assessing the metabolically active live cells. The discrepancy between the results might be attributed to the technique sensitivity of piezosurgery in cutting the bone with different densities. If a high amount of pressure is used for bone cutting or the flow of the fluid for irrigating the area is low during the cutting process, there is a high risk of thermal and mechanical trauma.^10^ Therefore, less osteogenic potential of harvested autogenous bone grafts could be expected.

Although experimental animal studies for determining cell viability can be helpful in evaluating the osteogenic potential of the graft, it cannot indicate the therapeutic success of augmentation surgery. Therefore further studies are required on the impact of harvesting technique on the process of graft consolidation.

## Conclusion

According to the results of present study, it can be expected that autogenous bone grafts have viable cells. With regard to limitations of the study, it can be concluded that autogenous bone grafts harvested with a manual instrument and a piezoelectric device would provide more viable cells.

## Acknowledgments

The head of department provided general support.

## Authors’ contributions

Neda Rastegarfard developed the original idea and the protocol, abstracted and analyzed the data and was the grantor; Janet Moradi Haghgoo contributed to the development of the protocol.

## Funding

This study was supported in part by the dental school of Hamadan University of Medical Sciences

## Competing interests

The authors declare no competing interests with regards to the authorship and/or publication of this article.

## Ethics approval

Ethical approval for the experiment was obtained from the Ethics Committee in Animal Research of Hamadan University of Medical Sciences under the code IR.UMSHA.REC.1393.10.214
